# Typical Monoterpenes as Insecticides and Repellents against Stored Grain Pests

**DOI:** 10.3390/molecules21030258

**Published:** 2016-02-23

**Authors:** Suelen L. Reis, Anieli G. Mantello, Jeferson M. Macedo, Erica A. Gelfuso, Cássio P. da Silva, Ana L. Fachin, Alexandre M. Cardoso, Rene O. Beleboni

**Affiliations:** 1 Unidade de Biotecnologia, Universidade de Ribeirão Preto, Ribeirão Preto, São Paulo 14096-900, Brazil; suelen.lorenzato@gmail.com (S.L.R.); amantello@yahoo.com (A.G.M.); jeferson2m@yahoo.com.br (J.M.M.); ericagelfuso@yahoo.com.br (E.A.G.); ksim_ksim@hotmail.com (C.P.S.); afachin@unaerp.br (A.L.F.); 2 Instituto Federal de Educação, Ciência e Tecnologia de São Paulo (IFSP), Barretos, São Paulo 14781-502, Brazil; amcardoso@ifsp.edu.br

**Keywords:** essential oils, *Callosobruchus maculatus*, fumigant, pest management, *Sitophilus zeamais*

## Abstract

Five monoterpenes naturally occurring in essential oils were tested for their insecticidal and repellent activities against the bruchid beetle *Callosobruchus maculatus* and the maize weevil *Sitophilus zeamais*. The monoterpenes were highly efficient as inducers of mortality or repellency against both insect species. They were more efficient in their fumigant activity against *C. maculatus* than against *S. zeamais*, while this profile of action was inverted when considering the repellent activities. Eugenol was one the most effective fumigants against both insects and one the most effective repellent against *C. maculatus*, while citronellal and geranial were one the most effective repellents against *S. zeamais*. Functional and positional isomerism of the monoterpenes pairs appears to exert little or no influence on theirs effects, especially in case of repellency. The validation of the insecticidal/repellent efficacy of isolated monoterpenes may permit a more advantageous, rapid, economic and optimized approach to the identification of promising oils for commercial formulations when combined with ethnobotanical strategies.

## 1. Introduction

Crop pests are one of the most important factors contributing to the decrease in productivity of different agricultural crops. These pests can attack during different stages of the food production process, affecting crops in the field or stored grains and causing major economic losses [1]. Therefore, pest management is crucial for the longevity and quality maintenance of food and related agricultural products, especially stored products since cumulative production costs are higher [1,2].

One of the most important pests attacking stored grains in Brazil and in the world are the cowpea seed beetle *Callosobruchus maculatus* (Fabr.) (Coleoptera: Bruchidae) and the maize weevil *Sitophilus zeamais* Motschulsky (Coleoptera: Curculionidae). These pests are particularly important due to wide range of crops attacked, higher economic losses and problems in their management. The management of these pests can be difficult since infestation with adult insects occurs throughout the entire crop growth period and is characterized by a high degree of inbreeding between different insect populations [3,4]. Today, *C. maculatus* and *S. zeamais* are the most important pests associated with stored cowpea (*Vigna unguiculata* L.) and maize (*Zea mays* L.), respectively [5,6]. Both insect species are commonly used as model organisms in biological research, especially to screen for new insecticides and repellents, because of their short generation interval and effortless maintenance [3,4].

Botanical pesticides are considered interesting alternatives to synthetic pesticides since they are potentially safer due to their rapid biodegradation and consequent short persistence in the environment. Furthermore, these pesticides are usually less expensive and less likely to induce pest resistance, especially when used as phytocomplexes in which a mixture of active compounds has different complementary modes of action on different molecular targets and insect stages of development [7,8].

Within this context, essential oils of plants are an interesting source of new botanical pesticides, especially because they are rich in monoterpenes [9]. Indeed, several monoterpenes have been shown to have remarkable insecticidal and repellent activities, including against *C. maculatus* and *S. zeamais*. Several studies have demonstrated these properties for monoterpenes such as cineol, limonene, terpinolene, and thymol, among others [10,11]. However, no direct insecticidal or repellent activity against *C. maculatus* and *S. zeamais* has been reported for other monoterpenes. Further investigation of these activities involving a larger number of different chemical structures is important, especially when considering the critical contribution of structure-activity relationship studies to the design and development of new biotechnological compounds such as safer and less expensive pesticides.

The aim of the present study was to evaluate the insecticidal and repellent activity of typical monoterpenes (geraniol, geranial, (±) citronellal, citronellol and eugenol) against the stored grain pests *C. maculatus* and *S. zeamais*. The monoterpenes were chosen based on their insecticidal and repellent potential, scientific originality, easy availability, and wide occurrence in essential oils of plants.

## 2. Results and Discussion

**Figure 1:**
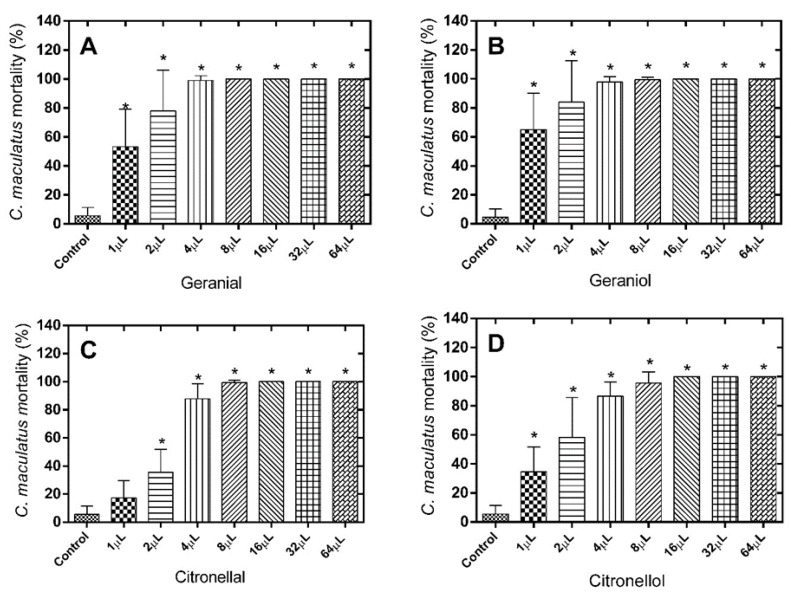

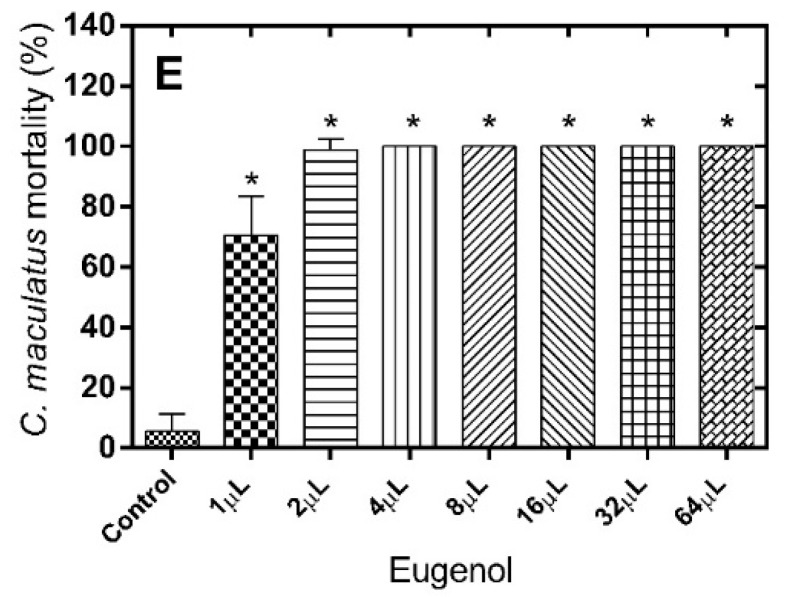
Mortality (%) of *C. maculatus* 24 h after exposure to the alimentary substrate treated with different monoterpene doses. (**A**) Geranial; (**B**) Geraniol; (**C**) Citronellal; (**D**) Citronellol; (**E**) Eugenol. * Significantly different compared to control, test (*p* < 0.05).

The results of the mortality tests show considerable effectiveness of the different monoterpenes against the insects tested, especially against *C. maculatus*. With respect to the number of dead *C. maculatus* after treatment with geranial (LD_50_ = 0.9832 µL), significant differences were observed for all doses tested when compared to the control group [F(7.71) = 56.22; *p* < 0.0001] (Figure 1A). The same was observed for the treatments with geraniol (LD_50_ = 0.7140 µL) [F(7.71) = 53.79; *p* < 0.0001] (Figure 1B), citronellal (LD_50_ = 2.261 µL) [F(7.71) = 217.8; *p* < 0.0001] (Figure 1C) (exception only for the dose of 1 µL), citronellol (LD_50_ = 1.534 µL) [F(7.71) = 76.54; *p* < 0.0001] (Figure 1D), and eugenol (LD_50_ = 0.9473 µL) [F(7.71) = 53.85; *p* < 0.0001] (Figure 1E).

**Figure 2:**
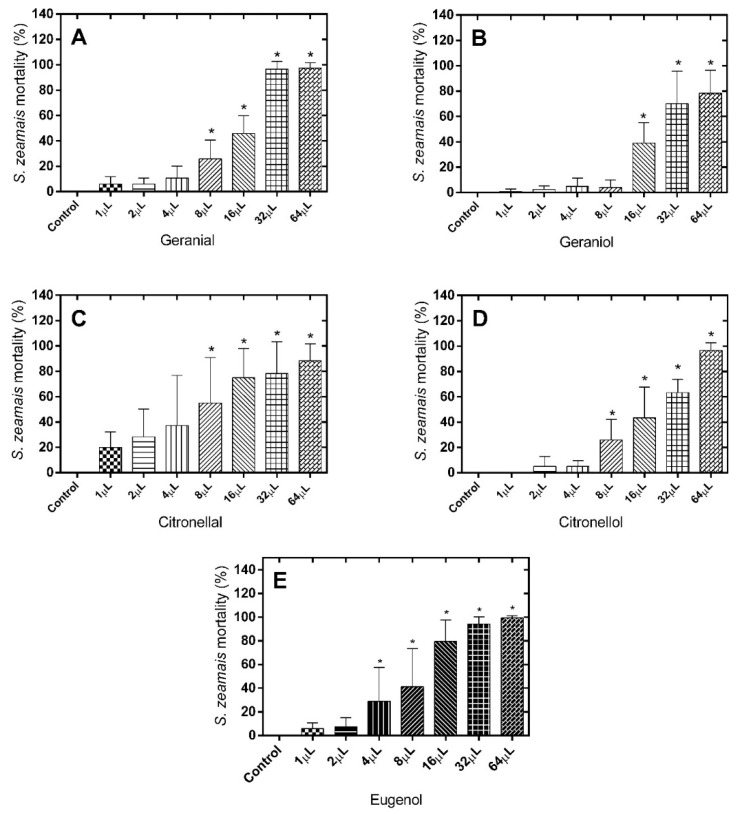
Mortality (%) of *S. zeamais* 24 h after exposure to the alimentary substrate treated with different monoterpene doses. (**A**) Geranial; (**B**) Geraniol; (**C**) Citronellal; (**D**) Citronellol; (**E**) Eugenol. * Significantly different compared to control, test (*p* < 0.05).

Regarding the number of dead *S. zeamais* after treatment with geranial (LD_50_ = 14.26 µL), only doses higher than 8 µL resulted in significant differences when compared to the control group [F(7.71) = 127,2; *p* < 0.0001] (Figure 2A). For geraniol (LD_50_ = 22.60 µL), only doses higher than 16 µL [F(7.71) = 39.92; *p* < 0.0001] (Figure 2B) was effective.

Similar results to those observed for geranial were obtained for the citronellal/citronellol pair (LD_50_ = 6.112 µL and 18.67 µL, respectively), with the occurrence of a significant effect only for doses higher than 8 µL (Figure 2C,D). In the case of eugenol (LD_50_ = 8.172 µL), the fumigant effect was already observed at a dose of 4 µL (Figure 2E). Treatment with the different monoterpenes exhibited a clearer dose-dependent profile of fumigant effect against *S. zeamais* than in case of *C. maculatus*.

Despite the proven effectiveness of the different monoterpenes against the two insect species tested; indistinctly considering LD_50_ and the generic profile of results, it can be stated that the terpenes tested clearly exhibited a greater fumigant effect against *C. maculatus* than against *S. zeamais*. These differences in effect may be due to the type of alimentary substrate used for maintenance of the test. In this respect, *C. maculatus* uses cowpea (*V. unguiculata* L.) as a substrate, while *S. zeamais* requires alimentary paste. These substrates differ in terms of the levels of humidity, rugosity and porosity, factors that can affect adhesion between the monoterpene/grain and monoterpene volatility. Nevertheless, these differences in fumigant potential can be better explained by differences in the sensitivity/resistance of the insects to the compounds tested as a result of their strict biological differences. They differ completely in genera, differing then in feeding parameters, exoskeleton, enzymatic systems, neurotransmission and olfactory reception [12,13,14]. Within this context, all compounds are effective against both insects, but are more selective for *C. maculatus* than *S. zeamais*. Considering the two species, eugenol is one of the most effective insecticides despite a slightly greater effectiveness of geraniol against *C. maculatus* which, on the other hand, is compensated for by its lower effectiveness against *S. zeamais*.

The functional isomerism of aldehyde and alcohol groups between the geranial/geraniol and citronellal/citronellol pairs does not seem to exert an important influence on the fumigant potential of the monoterpenes, since the LD_50_ values and especially the general profile of the results did not differ so much within each pair against *S. zeamais* or, especially, against *C. maculatus* (Figure 1 and Figure 2). The exception here is about the pair citronellal/citronellol against *S. zeamais*, in which the LD_50_ for citronellal was about three-fold lower compared with citronellol. On the other hand, positional isomerism appears to have a more important influence, since when considered specifically the values of LD_50_, geranial and geraniol presented LD_50_ about two-fold lower than in case of citronellal and citronellol, respectively, against *C. maculatus.* It is similar for the pair geranial and citronellal in which the LD_50_ is two-fold higher for the first compound against *S. zeamais*. (Figure 1 and Figure 2).

Table 1 and Table 2 show the repellent effects of the different monoterpene doses against *C. maculatus* and *S. zeamais*, respectively. A clear dose-dependent profile of the repellent effect could not be established for either insect or any of the monoterpenes tested. This fact permits comparison of the different doses of the compounds based on overall mean percent repellency.

Geranial exerted a significant repellent effect against *C. maculatus* at doses higher than 4 μL, with preference indices of −0.34 to −0.51. No dose-dependent profile was also observed for geraniol, which exhibited greater repellency at doses of 8 μL (−0.61), 1 μL (−0.57), and 64 μL (−0.48). Citronellal exerted a significant repellent effect against *C. maculatus* at all doses tested, with preference indices of −0.16 to −0.60. Similar results were obtained for citronellol, with repellency indices ranging from −0.13 to −0.58, except for the dose of 2 μL which was inefficient as a repellent (Table 1).

It is possible to assume that functional and positional isomerism exerts no influence on monoterpenes repellency effectiveness. This statement is based on the general profile of results, on the repellency performance exhibited by each isomer pairs (function and position) at a single dose and on the overall mean percent of repellency exhibited by them along the range of doses. The overall mean percent repellency at all doses of each compound ranged from 60 to 70% for the geranial (63.33% ± 9.5%)/geraniol (69.50% ± 8.7%), citronellal (67.50% ± 7.0%)/citronellol (66.50% ± 9.7%), geranial (63.33% ± 9.5%)/citronellal (67.50% ± 7.0%), and geraniol (69.50% ± 8.7%)/citronellol (66.50% ± 9.7%) pairs, with no significant differences between results ( *p* < 0.05) (Table 1). When pairs (function or position) were compared at the same dose, the only significant difference for functional pairs was observed between geranial/geraniol and geranial/citronellal at dose of 2 μL (Table 1) and between geraniol and citronellol at the doses of 2 and 8 μL (Table 1). It is a very discrete correlation in view of the enormous universe of possible comparison between pairs. Eugenol exhibited significant effectiveness as a repellent at all doses tested (preference indices ranging from −0.30 to −0.76) showing the highest overall mean percent repellency at all doses tested (75.63% ± 7.0%) compared to the other monoterpenes (Table 1).

**Table 1:** Repellent effect of different monoterpene doses against *C. maculatus* according to the percentage of repellency and preference index (PI).

Treatment	Adults Repelled (%) (*C. maculatus*)	PI
Geranial/Geraniol (1 µL)	51.67 ± 17.85/78.33 ± 15	−0.03 N/−0.57 R
Geranial/Geraniol (2 µL)	50.00 ± 10.38 ^a,b^/58.89 ± 8.85 ^a,b^	−0.00 N/−0.18 R
Geranial/Geraniol (4 µL)	67.22 ± 5.96/67.78 ± 14.16	−0.34 R/−0.36 R
Geranial/Geraniol (8 µL)	70.56 ± 14.24/80.56 ± 12.61 ^b^	−0.41 R/−0.61 R
Geranial/Geraniol (16 µL)	61.11 ± 19.32/58.20 ± 18.67	−0.22 R/−0.16 R
Geranial/Geraniol (32 µL)	67.22 ± 13.01/68.89 ± 13.64	−0.34 R/−0.38 R
Geranial/Geraniol (64 µL)	75.56 ± 18.44/73.89 ± 9.93	−0.51 R/−0.48 R
**Mean between all doses of each compound**	63.33 ± 9.5/69.5 ± 8.7 *	---------------------
Citronellal/Citronellol (1 µL)	57.78 ± 17.69/66.11 ± 14.6	−0.16 R/−0.32 R
Citronellal/Citronellol (2 µL)	80.00 ± 11.91 ^b^/52.22 ± 9.93 ^b^	−0.60 R/−0.04 N
Citronellal/Citronellol (4 µL)	68.33 ± 11.99/63.33 ± 16.0	−0.37 R/−0.27 R
Citronellal/Citronellol (8 µL)	68.33 ± 11.45/56.67 ± 15.81 ^b^	−0.37 R/−0.13 R
Citronellal/Citronellol (16 µL)	63.89 ± 10.54/78.89 ± 8.58	−0.28 R/−0.58 R
Citronellal/Citronellol (32 µL)	71.11 ± 11.93/73.89 ± 13.41	−0.42 R/−0.48 R
Citronellal/Citronellol (64 µL)	63.33 ± 13.46/75.00 ± 7.9	−0.27 R/−0.50 R
**Mean between all doses of each compound**	67.53 ± 7.0/66.7 ± 9.7 *	---------------------
Eugenol (1 µL)	77.22 ± 11.75	−0.54 R
Eugenol (2 µL)	72.78 ± 12.52	−0.46 R
Eugenol (4 µL)	73.89 ± 12.93	−0.48 R
Eugenol (8 µL)	65.00 ± 12.74 *	−0.30 R
Eugenol (16 µL)	79.44 ± 12.85	−0.59 R
Eugenol (32 µL)	73.33 ± 7.5	−0.47 R
Eugenol (64 µL)	87.78 ± 12.0	−0.76 R
**Mean between all doses of eugenol**	75.63 ± 7.0 *	---------------------

* Significantly different compared to control, test (*p* < 0.05); ^a^ compared to differ significantly between function isomers test (*p* < 0.05); ^b^ significantly different compared between position isomers test (*p* < 0.05); PI = Preference Index; Rating: R = repellent; N = neutral.

Geranial and geraniol exerted a significant repellent effect against *S. zeamais* at all doses tested. The preference indices ranged from −0.33 to −0.92 for geranial and from −0.17 to −0.83 for geraniol, without the observation of a clear dose-response profile effect. Citronellal was also effective at all doses, with preferences indices of −0.72 to −0.95, while citronellol was ineffective at the lowest dose (1 μL) and showed indices of −0.23 to −0.68 after the second dose (Table 2). Despite the slight discrepancy between the citronellal/citronellol pair, with a mean percent repellency of 92.16% ± 3.9% and 72.99% ± 12.5%, respectively, functional or positional isomerism does not seem to influence the repellency results reported here, as seen in the interpretations above. Indeed, the functional pair geranial/geraniol exhibited overall mean percent repellency of 87.75% ± 10.3% and 83.5% ± 12.1%, respectively. The positional pair geranial/citronellal exhibited overall mean percent repellency of 87.75% ± 10.3% *vs.* 92.16% ± 3.9% and geraniol/citronellol of 83.50% ± 12.1% *vs.* 72.99% ± 12.5% (Table 2). Comparison according to isomer pair (function or position) revealed results similar to those obtained for *C. maculatus*, with a very discrete or zero correlation between functional or positional isomers and the repellent effect observed (Table 2). The few significant differences between functional isomers were exactly observed between citronellal and citronellol at the lower doses. Some significant differences between positional isomers occurred between the geranial and citronellal pair only at the doses of 1 and 16 μL and between the geraniol and citronellol pair only at the dose of 2 μL (Table 2). The mean percentage of repellency of eugenol was only 63.81% ± 13.14%. Eugenol did not exhibited repellency for all dose tested. In fact, eugenol is not repellent at both the lowest and the highest doses, while it is repellent at intermediate doses. In this point is important to consider the well-known attractant effect of eugenol for some pests, particularly flies. For *S. zeamais*, citronellal and geranial exhibit a better repellent effectiveness than the other monoterpenes (Table 2).

**Table 2:** Repellent effect of different monoterpene doses against *S. zeamais* according to the percentage of repellency and preference index (PI).

Treatment	Adults Repelled (%) *(S. zeamais)*	PI
Geranial/Geraniol (1 µL)	66.67 ± 14.0 ^b^/58.33 ± 8.1	−0.33 R/−0.17 R
Geranial/Geraniol (2 µL)	96.67 ± 2.58/91.67 ± 5.16 ^b^	−0.93 R/−0.83 R
Geranial/Geraniol (4 µL)	95.83 ± 4.91/77.50 ± 14.95	−0.92 R/−0.55 R
Geranial/Geraniol (8 µL)	91.67 ± 5.16/89.17 ± 9.17	−0.83 R/−0.78 R
Geranial/Geraniol (16 µL)	92.50 ± 8.16 ^b^/90.00 ± 4.47	−0.85 R/−0.80 R
Geranial/Geraniol (32 µL)	84.17 ± 10.68/90.83 ± 7.36	−0.68 R/−0.82 R
Geranial/Geraniol (64 µL)	86.67 ± 9.3/87.50 ± 15.0	−0.73 R/−0.75 R
**Mean between all doses of each compound**	87.74 ± 10.3 */83.57 ± 12.1 *	---------------------
Citronellal/Citronellol (1 µL)	85.83 ± 5.48 ^a,b^/50.00 ± 21.21 ^a^	−0.72 R/−0.00 N
Citronellal/Citronellol (2 µL)	90.98 ± 8.2 ^a^/61.67 ± 8.7 ^a,b^	−0.82 R/−0.23 R
Citronellal/Citronellol (4 µL)	93.33 ± 12.5/80.00 ± 10.5	−0.87 R/−0.60 R
Citronellal/Citronellol (8 µL)	97.50 ± 4.18/80.00 ± 12.65	−0.95 R/−0.60 R
Citronellal/Citronellol (16 µL)	95.83 ± 5.48 ^b^/80.83 ± 9.7	−0.92 R/−0.62 R
Citronellal/Citronellol (32 µL)	90.83 ± 10.2/74.26 ± 4.91	−0.82 R/−0.49 R
Citronellal/Citronellol (64 µL)	90.83 ± 5.48/84.17 ± 11.43	−0.82 R/−0.68 R
**Mean between all doses of each compound**	92.12 ± 3.9 ^a,^*/72,99 ± 12.5 ^a,^*	---------------------
Eugenol (1 µL)	43.33 ± 19.66	0.13 A
Eugenol (2 µL)	75.00 ± 14.83	−0.50 R
Eugenol (4 µL)	54.17 ± 13.93	−0.08 N
Eugenol (8 µL)	73.33 ± 15.38	−0.47 R
Eugenol (16 µL)	69.17 ± 26.15	−0.38 R
Eugenol (32 µL)	77.50 ± 2.73 *	−0.55 R
Eugenol (64 µL)	54.17 ± 22.23	−0.08 N
**Mean between all doses of each compound**	63.81 ± 13.14 *	---------------------

* Significantly different compared to control, test (*p* < 0.05); ^a^ compared to differ significantly between function isomers test (*p* < 0.05); ^b^ significantly different compared between position isomers test (*p* < 0.05); PI= Preference Index; Rating: R = repellent; N = neutral; A = attractive.

Despite the suggestion that functional or positional isomerism exerts little or no influence especially in case of repellent potential of the monoterpenes tested, some technical limitations of this descriptive-preliminary interpretation should be considered, including the need for a wider dose range and a greater representativeness of the functional or positional isomer pairs, represented here only by the geranial/geraniol, citronellal/citronellol, geranial/citronellal and geraniol/citronellol pairs.

Considering the general profile of the results, as well as LD_50_ values, preference indices and percent repellency, it can be suggested that most of the monoterpenes were more effective fumigants against *C. maculatus* than against *S. zeamais*, but in general more effective repellents for *S. zeamais* than for *C. maculatus*. Of course, since *S. zemais* is more repelled it avoids these insects to progress to the death when in comparison to *C. maculatus*. Again, these differences between the results obtained for *S. zeamais* and *C. maculatus* can be explained by the biological differences of the insects which belong to completely different genera and species, although both are pests of stored grains. Differences in feeding patterns, exoskeleton and enzymatic and neurotransmission systems, as well as marked differences in the olfactory systems/receptors used to process volatile chemical information from the environment (odorant receptors, specific gustatory receptor proteins and ionotropic receptors, as well as peripheral sensory reception and signal transduction), may at least in part explain the different results. Although ubiquitous in some cases, many of these biological systems cited above are species specific [13,14,15].

Monoterpenes and other compounds are found at different concentrations and proportions in different essential oils. The synergistic or complementary activities of different compounds present in the same oil play an extremely important role in the final insecticidal and/or repellent activity. Within this context, minor compounds can support or regulate the activity of major compounds or, less commonly, vice-versa [12,16]. In some cases, this fact can favor the choice of the intact essential oil of a plant for the final insecticide/repellent formulation instead of an isolated compound, with different mechanisms of action contributing to the final effect on the target organism. The choice of the oil can be guided by the knowledge of the insecticidal and/or repellent activity of the isolated compounds, as provided in the present study for the typical monoterpenes tested. Thus, the validation of the isolated active ingredients for different effects, including insecticidal and repellent activity, may provide a more advantageous, rapid, economic, effective and optimized approach to the identification of promising oils for commercial formulations than the conventional strategy. The latter consists of prospecting an enormous number of oils for pharmacological activities of interest and the subsequent study of the active ingredients responsible for the action observed. In other words, once the action of a given active ingredient is validated, oils rich in this compound can become potentially optimized candidates for new studies and for the elaboration of commercial formulations, including insecticides and repellents.

The main families of essential oil-producing plants studied for insecticidal and/or repellent activity include Lamiaceae, Labiatae, Lauraceae, Asteraceae, Myrtaceae, and Cupressaceae. These plants may be optimal sources for the choice of pharmaceutical phytocomplexes to be used as insecticides/repellents based on the knowledge of isolated active ingredients with activity against defined target insects [10,17,18]. Once the insecticidal activity of certain active ingredients is known, it is possible to search for these substances in essential oils from different plants for the production of an effective pharmaceutical phytocomplex. Plants of the genera *Cymbopogon* spp., *Eucalyptus* spp. and *Ocimum* spp. are the most widely studied as sources of insecticidal and/or repellent oils and are also the ones most commonly found in commercial formulations for this purpose [18,19]. Characteristically, these genera are rich in monoterpenes, including eugenol, citronellal and citronellol also investigated in this study [18].

Indeed, essential oils of *Eucalyptus benthamii*, *E. dunnii* and *E. saligna* exerted insecticidal activity against *S. zeamais*, with LD_50_ values of 25.03, 37.93 and 121.09 µL, respectively, confirming the popular use of eucalyptus as an insecticide for different purposes. All three oils were also significantly effective as repellents for *S. zeamais* [20]. Tests against *S. zeamais* using plants of the genus *Ocimum* ssp. also showed good activity. For example, the oil of *Ocimum gratissimum* killed 98% of adult weevils after 24 h of exposure in fumigation tests [21]. Curiously, there is no or only very little information about tests involving species of the genus *Cymbopogon* against *S. zeamais*, which could be an attractive alternative considering the great potential of the oil as insecticide and repellent, particularly because it is rich in monoterpenes [18]. Tests against *C. maculatus* using plants of the genus *Eucalyptus* ssp. have also shown good activity, with the oil of *Eucalyptus citriodora* killing 100% of adults of this insect after 48 h of exposure in fumigation tests [22]. Izakmehri *et al.* [23] reported LD_50_ values of 56.7 and 26.1 µL/L air, respectively, after 12 and 24 h of exposure of *C. maculatus* to the oil of *E. camaldulensis*. Tests involving *C. maculatus* treated with different doses of essential oils of *Ocimum basilicum* L. and *Ocimum gratissimum* L. for the control of adults also yielded excellent results [24]. These studies highlight the importance of the potential use of essential oils and/or isolated monoterpenes to management of these insects.

Considering the potential use of monoterpenes and oils as insecticides/repellents, as well as the advantages of botanical pesticides and the importance of *C. maculatus* and *S. zeamais*, the present study contributes to the identification of new agents of biotechnological interest which could be used in commercial formulations that are less expensive, safer and more effective than currently available ones.

## 3. Experimental Section

### 3.1. Chemicals

All monoterpenes tested (geraniol, geranial, (±) citronellal, citronellol and eugenol) were purchased from Sigma-Aldrich (São Paulo, Brazil), with their respective datasheet containing the certificate of analysis attesting for purity and chemical identity.

### 3.2. Insects

The bruchid beetle *C. maculatus* and the maize weevil *S. zeamais* were maintained in stock cultures as described by Toscano *et al.* [25], with minor modifications. The culture conditions, which were the same for all experiments, were: 25 ± 2 °C, light-dark cycle of 12 h, and 60%–80% relative humidity. Unsexed weevils or beetles used in the fumigation and repellency assays were about 48–72 h post-eclosion.

### 3.3. Fumigant Toxicity Assay

The fumigant toxicity tests were carried out according to the procedures described by Mossi [20], with modifications. Briefly, 20 adult insects were placed in the screw cap of a glass vial (6.1 cm in diameter and height of 2.1 cm) containing 10 g of small pieces (homogeneous in weight and length) of alimentary paste for *S. zeamais* or cowpea (*V. unguiculata* L.) for *C. maculatus*. For the different treatments, different volumes of each monoterpene tested (1–64 µL) were uniformly dispersed in the respective alimentary substrate. The vials were then sealed until counting of the insects at the end of the experiment. The same experimental arrangement was used for the control groups, except for the addition of monoterpenes. The percentage of insect mortality was determined after 24 h of the experiment using the formula of Abbott [26]. LD_50_ values were calculated by non-linear regression. Each experiment was performed in triplicate and repeated at least two (*C. maculatus*) and three times (*S. zeamais*).

### 3.4. Repellency Assay

These experiments were carried out as described by Mossi [20] and Pettersson [27], with modifications. The repellent effects of the monoterpenes were assessed using an arena apparatus (30 × 30 cm) composed of five circular plastic boxes referred to as A, B, C, D and E (6.1 cm in diameter and 2.1 cm in height). The central box (A) was interconnected to the other boxes (B, C, D and E) by plastic tubes (0.5 cm in open diameter and 10 cm in length) for free choice of the 20 unsexed insects to access treated or untreated alimentary substrate. In all boxes, 10 g of small pieces (homogeneous in weight and length) of alimentary paste for *S. zeamais* or cowpea (*V. unguiculata* L.) for *C. maculatus* was added, except for the central box (A). For the different treatments, different volumes of each monoterpene tested (1–64 μL) were uniformly dispersed in the respective alimentary substrate in boxes B and D arranged in a diagonal orientation, while boxes C and E, also in a diagonal orientation, served as controls (without monoterpenes). The number of insects per box was determined 24 h after the beginning of the experiment. The preference index (PI) was determined as follows [28]: PI = It − Ic/It + Ic, where *It* is the percentage of insects in the boxes treated with the monoterpenes and Ic is the percentage of insects in the control boxes (without monoterpene). The preference index was classified as follows: −1.00 to −0.10, repellent monoterpene; −0.10 to +0.10, neutral monoterpene, and +0.10 to +1.00, attractant monoterpene. Each experiment was performed in triplicate and repeated at least two (*C. maculatus*) and three times (*S. zeamais*). The percentage of repellency per treatment at each dose was calculated and used for the comparison between the different function and position isomers. The overall mean percentages of repellency obtained for all doses of each compound were also compared.

### 3.5. Statistical Analysis

All statistical analysis was performed using ANOVA followed by the Tukey’s test (*p* < 0.05) using the GraphPad Prism 5.1 program (GraphPad Software, San Diego, CA, USA, version 5.01).
